# A Lean UX Process Model for Virtual Reality Environments Considering ADHD in Pupils at Elementary School in COVID-19 Contingency

**DOI:** 10.3390/s21113787

**Published:** 2021-05-30

**Authors:** Héctor Cardona-Reyes, Jaime Muñoz-Arteaga, Klinge Villalba-Condori, María Lorena Barba-González

**Affiliations:** 1 CONACYT Research Fellow, Center for Research in Mathematics, Zacatecas 98160, Mexico; 2 Departamento de Sistemas de Información, Universidad Autónoma de Aguascalientes, Aguascalientes 20131, Mexico; jaime.munoz@edu.uaa.mx; 3 Vicerectorado de Investigación, Universidad Católica de Santa María, Arequipa 1350, Peru; kvillalba@ucsm.edu.pe; 4 Center for Research in Mathematics, Quantum Knowledge City, Zacatecas 98160, Mexico; maria.barba@cimat.mx

**Keywords:** lean UX, user experience, virtual environments, ADHD

## Abstract

Today, the world is experiencing the COVID-19 health contingency, which prevents people from being exposed to one another and restricts physical contact. Under this context, the use of technology has become an essential tool to face the challenges of daily life, and virtual reality can be an alternative in the development of solutions that effectively support the acquisition of learning skills and knowledge transmission through the execution of tasks designed by multi-disciplinary groups. In addition, it can encourage the user to continue with the acquisition of learning skills in a friendly and fun way in a health and education context. This work proposes the use of virtual reality environments as an alternative to support the learning process in children with special educational needs such as Attention Deficit Hyperactivity Disorder (ADHD) and other associated disorders that occur in basic education. These proposed reality environments are designed under the Lean UX process model and their contents are designed according to expert therapeutic guidelines. As a result of this proposal, a case study is presented in which the user experience is evaluated through the use of an interactive environment to support the special educational needs of elementary school children attending an educational institution in Mexico.

## 1. Introduction

The world has been experiencing a health contingency since January 2010, originating from a disease called COVID-19, caused by a virus called SARS-CoV-2 [1,2]. Today, the pandemic continues, and the consequences worldwide have been catastrophic in different sectors of health, economy, industry, and education, among others [3]. As the COVID-19 pandemic ravages the world, it is essential to address the educational needs of children and youth during the crisis [4]. Mental health and education is an important area to highlight, and can contribute to the education sector at the basic school level with technology-based approaches that can be adapted to containment measures established by governments as a contagion prevention measure.

Children and adolescents are part of a sector that has been affected by this pandemic in terms of mental health, education, and, in some cases, contagion. Among the most vulnerable sectors are the elderly, the chronically ill, and children. In the case of the latter, although not considered to be affected, they are considered to be carriers of the virus [5,6].

Globally, according to the World Health Organization (WHO) [7], as of 24 March 2021, a total of 123,902,242 confirmed cases and more than 461,606 new cases had been reported, with an estimated total of 2,727,837 deaths worldwide. Among the most affected countries are the United States with 29,592,831 cumulative cases, Brazil with 12,047,526 cases, India with 11,734,058 cases, Mexico with 2,197,160 cases and 198,239 cumulative deaths, and Peru with 1,472,790 cases and 50,339 cumulative deaths.

Education is a basic right that allows children and adolescents to acquire skills and knowledge necessary to develop in the social context as adults [8] and it is important that, during contingencies, this right is given continuity by seeking alternatives to achieve the objective of education. The most important impact worldwide derived from the pandemic is the closure of schools since it causes the loss of learning, school dropouts, and greater inequity, without taking into account the adverse effects such as the economic crisis in households, which results in a low educational demand [9].

The sudden confinement measures suggested a change for all those involved in the education sector, schools, teachers, students—children and adolescents—to adopt information and communication technologies as an adaptive tool to the new ways of working, both for teachers and children’s learning. Now, in the area of special education, in addition to this sudden change, children suffer a stagnation because they require specialized attention from teachers and specialists. For them, not only were classes stopped, but also the attention received to improve symptoms of disorders that some students suffer from, such as ADHD, autism, Asperger, etc.

In this sense, new approaches can be proposed through the use of technologies such as virtual reality to strengthen and complement the teaching–learning system in the basic and secondary education stage, in the care in external services of patients with some special education disorder or disability, even with the teachers or tutors themselves. Since education is virtual, information and communication technologies are appropriate tools to enhance development and learning in these times of this pandemic.

This work is composed of eight sections. Section 2 presents proposals where ICT, mainly virtual reality, has been a support for educational processes. Section 3 presents a literature review of the context of ADHD and the advantages of virtual reality and how it can be used in times of a pandemic. Section 4 proposes the Lean UX process model to produce user-centered virtual reality environments according to the needs of children with ADHD in a school context. Section 5 presents a case study under the Lean UX model, in which the virtual reality environment Attention VR is proposed as part of activities conducted in elementary schools. Section 6 presents the results obtained, the discussion is presented in Section 7, and finally, the conclusions and future work are presented in Section 8.

## 2. Related Work

Researchers in the field of education and health are constantly looking for new ways to effectively integrate ICT in educational processes and even seek these to support the treatment of various chronic disorders such as ADHD and others that affect the student’s educational environment. In this sense, one of the emerging technologies that has become more relevant today is Virtual Reality (VR), because in the educational field it can help students to acquire and develop meaningful learning, and VR can also be a support for the evaluation and rehabilitation of cognitive processes and functional abilities of students with ADHD [10,11].

Studies have been conducted such as the one by Rizzo et al. [12], where a VR system was proposed for the assessment and rehabilitation of attention processes, as these have a high incidence in ADHD clinical conditions. The proposed VR system provides a stimulus-controlled environment in which cognitive challenges are presented where there is precise control of auditory and visual stimuli (distractors). The VR system provides attention and distraction challenges within a valid environment for the user, such as a classroom.

Barba et al. [13] proposed the BRAVO project (Beyond the treatment of the Attention-deficit hyperactivity disorder), which consists of the development of an immersive, serious game aimed at improving the way young patients carry out their ADHD rehabilitation. It is mainly based on gamification and the serious games approach. In addition, this project allows the incorporation of wearable equipment and virtual and augmented reality devices. Among the advantages mentioned in this project are the improvement of the way patients carry out their therapy, the incorporation of personalized therapeutic processes, and support to therapists in the management of rehabilitation.

Other proposals such as that of Yang et al. [14] include games for children in virtual reality to support ADHD rehabilitation, such as exercise-based games that allow children to train balance and coordination and thus improve their cognition and intelligence. In his proposal, Yang presents a study in which a series of VR games focused on body coordination training were implemented with the aim of improving the cognitive capacity, abstract reasoning, and complex information processing of children with ADHD. In the case of Baumann et al.’s [15] proposal, it focuses on presenting virtual reality environments that help the consolidation of long-term memory, bringing benefits to the memory problems observed in children and adolescents with ADHD.

In Avila et al.’s [16] work, the focus was on using the serious games approach and Augmented Reality (AR) using a Kinect-based interface to present a prototype focused on providing support to cognitive-behavioral therapies for school-aged children with ADHD. As a result, this prototype was tested on 11 children with ADHD between the ages of 7 and 10 years. In addition, during this experiment, a therapist participated in the experiment and carried out his therapy in a traditional way using a memory game and then the prototype called ATHYNOS. Finally, the results obtained from this experiment indicate that the children showed great interest and motivation, together with the therapist’s satisfaction with the use of the prototype in his interventions with the children.

Machine learning technologies such as Deep Learning (DL) in conjunction with virtual reality have been part of proposals for the generation of diagnostic tools for ADHD. Wiguna et al. [17] proposed a DL model and a diagnostic tool for ADHD-VR for the diagnosis of this disorder, which can be used especially in those places with few resources and where there are no specialized personnel such as doctors, child psychologists, or pediatricians, so that they can follow up this type of disorders and can establish the teleconsultation. This proposal aims to be a digital diagnostic tool under a DL model in order to ensure reliable and early diagnosis of ADHD and provide evidence of the diagnosis to parents and specialists.

The current work focuses on a multidisciplinary approach, i.e., to involve the family, the institution of education, counselors, or psychologists together with technologists to incorporate solutions based on technology to assist the child from the early stages of the therapeutic process of suspicion and treatment of ADHD and provide interactive virtual reality environments according to the needs treatment and school context.

## 3. Background

### 3.1. ADHD in the School Context

Attention Deficit Hyperactivity Disorder (ADHD) is a chronic neurodevelopmental disorder more frequent in the school stage. Worldwide, there is a record that 5% of the population has this disorder, 10% of children between 4 and 7 years have an identification of ADHD, and on average, two or three children in a classroom may have this disorder [18]. In the United States, between 7% and 9% of the population under 18 years of age [19], and in Mexico, between 4% and 12% of the school population, is affected [20].

Symptoms are frequently detected by parents and teachers who are with the child, who perceive behaviors different from the rest of the children of their age; some of these symptoms must have appeared before seven years of age, must occur in two or more environments (school, family, etc.), and cause a clinically significant impairment of social, academic, or work activity. It is the clinician who diagnoses ADHD [21], and it is a neurodevelopmental disorder that occurs more frequently during the school stage. According to the Diagnostic and Statistical Manual of Mental Disorders-5 (DSM-5), its prevalence is estimated at 5%; it is more frequent in males than in females in a ratio of 2:1. (American Psychiatric Association [APA], 2013.

ADHD is classified into three subtypes [22]:

- Combined presentation: If Criterion A1 (inattention) and Criterion A2 (hyperactivity-impulsivity) are met during the last 6 months.
- Inattentive predominant presentation: If Criterion A1 is met but Criterion A2 (hyperactivity-impulsivity) is not met during the past 6 months.
- Predominantly hyperactive/impulsive presentation: If Criterion A2 (hyperactivity-impulsivity) is met and Criterion A1 (inattention) is not met during the last 6 months.

This work distinctly considers the three diagnostic classifications presented by the student: Predominantly inattentive, hyperactive/impulsive, or combined. In this sense, proposing virtual reality environments can be a tool for students to put their ideas and learning into practice, obtaining feedback in a short time, and even being able to be carried out in their homes. In this way, virtual reality environments help foster rapid interaction and real-time feedback, keep students active by performing activities on their own or in collaboration with other students, and, finally, allow teachers to have tools to measure student performance and provide feedback. This allows one to adapt to pandemic confinement measures through the use of accessible platforms and devices.

There are several investigations that explain the causes, with different explanatory models, from the biological to the motivational model of Barkley that proposes a deficiency at the motivational level in behaviors governed by rules [23]. Children with ADHD have difficulty inhibiting immediate responses, perseveration of responses in progress, and poor control of interference [24], meaning ADHD is understood to be a disorder of behavioral inhibition [25]. In the school context, a central axis lies in inattention and excessive motor activity, and attention is an essential requirement for proper cognitive functioning because it has two main functions: The first is to maintain the alertness of the cognitive system and the second is to select relevant information provided by the environment [26]. Furthermore, poor control and excessive motor activity exceeds normal limits according to their age and maturity level, they have the need to move constantly and lack body and emotional self-control, with consequences in their school performance, and impulsivity causes that children do not foresee the consequences of their actions, and the need to immediately satisfy their desire is seen in the absence of control. Children tend to interrupt, cannot be still, talk too much, do not wait their turn, do not follow directions and do not finish tasks, and constantly get up from their place [22]. At the cognitive level, it is manifested in the inaccuracy and deficit in processes of perception and the analysis of information in complex tasks [27].

### 3.2. Virtual Reality Environments

Virtual reality is a technology that is increasingly adopted in various fields of application, and although it has always been popularized in the field of video games, fields such as medicine and education are increasingly oriented towards the use of virtual reality devices as part of the improvement of their processes and user experience [28,29]. Virtual reality allows users to interact with 3D representations generated by a computer [30,31] and the user is able to experience an immersive environment [32], that is, the user can have the feeling of realism at all times by interacting with all elements of the system and receive visual and sensory feedback in real-time through additional input/output devices such as the headset, sensors, controls, etc. [33,34].

### 3.3. Lean UX

Lean UX is a technique preconized by Toyota’s manufacturing model that works in alignment with Agile development methods. Jeff Gothelf, the author of Lean UX [35], quoted “Lean UX is about bringing the true nature of a product to light faster, in a collaborative, cross-functional way that reduces the emphasis on thorough documentation while increasing the focus on building a shared understanding of the actual product experience being designed.” For this work, Lean UX has helped to develop educational digital resources to be more effective when it comes to achieving critical goals of inclusive educational application. Lean UX aims to reduce waste and provide value. In fact, Lean UX combines the solution-based approach of design thinking with the iteration methods that compound Agile [35].

## 4. Method

This section proposes a model inspired by the Lean UX process model for the production of user-centered interactive virtual reality environments with ADHD. The stages that compose the model are based on proposals for the new production of interactive systems [36].

**Figure 1:**
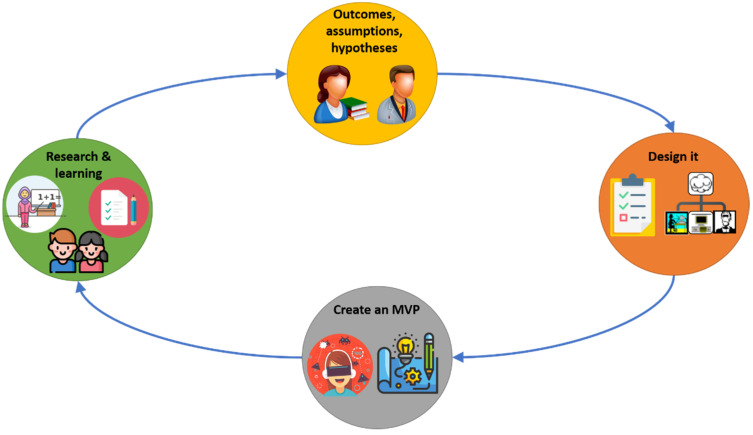
Lean UX Model for the production of virtual reality environments for users with ADHD (reprinted of Gothelf’s model) [35]).

The iterative Lean UX method of Figure 1 helps to design and craft user experiences. This method can be referred to as an agile UX (inspired from the Gothelf’s general Lean UX model [35]), and it allows to collect rapid-fire feedback from main educational actors (such as students, fathers, and teachers) in the hopes of making inclusive educational and assertive decisions. This feedback loops, and quick iterations also ensure that no data are left uncovered and no positive changes are left undone. The model of Figure 1 supports the production of learning environment prototypes in particular to cover different learning strategies that allow to attract attention and increase the study concentration required for students with ADHD. The feedback information is a key factor throughout the entire process that can make improvements to the user experience. As in HCI literature, it is necessary to ground the description of the new approach into a case study. Each phase of the proposed model of Figure 1 is described in detail below.

### 4.1. Outcomes, Assumptions, and Hypotheses

At this stage, it is necessary to define the outcomes, assumptions, and hypotheses for the production of new educational resources as well as the organization of human resources involved in the attention to children with ADHD, followed by the formation of a multidisciplinary team to learn, through evaluations and classroom dynamics, the use of materials for the tasks performed by the children. In fact, there is a great need for the assumptions in Lean UX without deliverables, and the focus is on how to produce changes to improve the product at the moment. Assumptions are necessary since they help to inform our hypotheses, which we will discuss shortly. The detection of ADHD and the evaluations for school monitoring together with the tasks carried out with the children allows the software analysts to design alongside the teachers and specialists, those that can be supported by virtual reality.

### 4.2. Design It

In the design stage, the elements obtained in the previous stage are transferred to a computational context to define the necessary software design components for virtual reality environments. The proposed Lean UX-centered model is considered a collaborative iterative process. There is good accountability and a faster design process. By bringing designers and non-designers together for co-creation, the yield of ideas is bigger and better than if it were solely done with individual contributors. In this way, everybody gets to design. In this stage, instructional designers are involved along with teachers, media, and subject matter specialists. The user tasks carried out in the sessions with the children allow the software analysts to design and propose ideas alongside the teachers and specialists, those that can be supported by using virtual reality. As a result of this stage, various assessments used by specialists in ADHD are obtained, the identification of tasks and materials that can be represented in virtual reality occurs, and a proposal for a platform is produced according to the learning needs of children and the user profile to which the proposed virtual reality environment will be directed.

### 4.3. Create a Minimum Viable Product (MVP)

Once the technological platform has been identified, along with the definition of tasks within the virtual reality environment as well as the interactions and processes of the user with the system, the software programmers gather the components and tools necessary for the construction of the prototype. Since the cycle is short in a Lean UX process, it lends itself to efficiency and speed. The focus for Lean UX is to ensure a minimum viable product (MVP) goes to real scenarios. Create the minimum, get it out, understand the reaction, iterate, and so on. In this sense, programmers can work together with multimedia designers to produce 3D models, sounds, and representations of objects that will be part of the scenario under which the user will carry out their tasks.

### 4.4. Research and Learning

Once you have a functional prototype with virtual reality, then it is possible to conduct a series of tests with final users ADHD. The aim is that these virtual reality environments produced are incorporated into the work sessions that teachers carry out with children and can be a support that gives continuity to the development of skills while they are in contingency conditions. The use of evaluations at this stage such as the User Experience Questionnaire (UEQ), System Usability Scale (SUS) [37], and AttrakDif [38], among others, will allow for an understanding of the perception and ease of use of virtual reality environments and the user experience, in addition to obtaining feedback information that allows one to know the behavior of children when immersed in virtual reality. In this stage, the feedback information is a key factor for the entire multidisciplinary team to make improvements and ensure the quality of virtual reality environments produced.

## 5. Case Study

The Mexican Ministry of Education has limited face-to-face activities in schools due to the COVID-19 pandemic [39] in order to avoid the risk of contagion in children. Therefore, the dynamics carried out in classrooms had to be adapted to an online modality and in some cases, semi presential and when the activity merits personal attention by teachers. These changes had an impact on those children who suffer from ADHD and other associated disorders because the attention they receive from teachers is personalized, and they focus on learning activities that help them mitigate educational lag.

In this case study, the participants constitute a multidisciplinary team made up of a team of a psychologist, a pedagogue in special education, a social worker, six teachers, and three technologists (a software tester, an analyst, and a programmer) who actively participate in the activities carried out at the school. The technologists were added to this case study for the development of the virtual reality environment. This team has attended to several children with a certain level of ADHD at an elementary school in Mexico, which works in the online and semi-presential modality due to the contingency limitations of the COVID-19 pandemic (see Supplementary Material).

The current work implemented the process model of Figure 1, where a virtual reality environment was designed and developed for elementary school children with special educational needs such as ADHD, among others.

### 5.1. First Iteration

The first iteration of the proposed process model was developed to identify the teachers’ requirements and the requirements of students that were attending the elementary school.

#### 5.1.1. Outcomes, Assumptions, and Hypotheses

**Figure A1:**
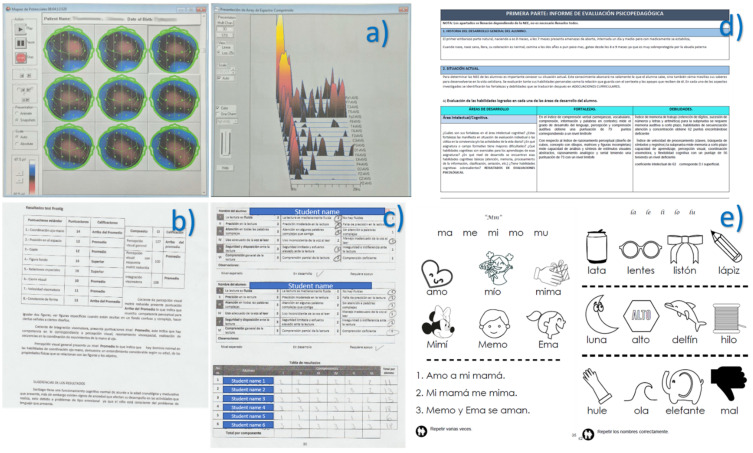
Evaluations for identification and follow-up in children with ADHD. (**a**) Digital electro-encephalogram with brain mapping. (**b**) Frostig test. (**c**) SisAt test. (**d**,**e**) Evaluation in children with intellectual disabilities and exercises taken from remote inclusion work booklet, by Unit for Regular Education, Mexico.

A first assumption is to have the means to know the end-user. To detect ADHD, there is no simple test since this disorder affects the brain and, in some cases, children need to be seen by doctors and psychologists who can diagnose whether a child shows certain signs of inattention in multiple environments for at least 6 months and if the parents or the child manifests a negative effect in their life [22]. Typically, these impairments in a school setting range from the child being easily distracted to having trouble waiting their turn or staying in their seat. During this case study, it was possible to learn about the different strategies that are carried out in elementary school to follow up on each child with ADHD. In particular, a case of a child whose ADHD was detected by means of a digital electroencephalogram (EEG) study was identified [40]. It is worth mentioning that this test is non-invasive and measures slow brain waves called theta waves and fast brain waves called beta waves, and the objective is to look for changes in brain patterns [41,42]. Other instruments used in elementary school (see Figure A1) as supports used by teachers and psychologists consist of evaluations such as the Frostig test that helps to identify delays in perceptual maturity in children with learning difficulties [43] and the test “Sistema de Alerta Temprana (SisAT)” [44] that helps teachers to perform early intervention actions in terms of support for reading, writing, and mental calculation, allowing to define school support strategies. In addition, pedagogues and teachers keep track of the activities with students with ADHD month-to-month, and materials such as booklets are used (Figure A1d,e), which contain exercises specifically selected to highlight the abilities of these students.

As a second assumption, we have considered that some ADHD students could be identified in an elementary school during the COVID-19 pandemic. The school currently serves a total of 114 primary-level children distributed in six school grades, of which 8 children were identified with ADHD, 2 with Asperger, and 12 with intellectual disabilities as presented in Table 1. In addition, the social worker and teachers obtained the parental consent of 16 children, of which 7 are children with ADHD and 9 children are regular for this case study.

The members of the team believed that identifying some virtual environments will achieve significant educational support in the sessions for attending to the students with ADHD presented in Table 1. An interesting answer to this hypothesis is described in detail in the next sub-sections.

#### 5.1.2. Design It

This section presents the design of the Attention-VR virtual reality environment under the proposed model developed for elementary school children and children with special educational needs such as ADHD, among others. The goal of Attention-VR is that the child can discover and interact with 3D objects in an immersive environment and make decisions to solve problems related to learning basic math. Table 2 specifies the instructional design required by teachers and educational experts, focused on the development of the areas of location, attention, motivation, structure, following instructions, motivation, and feedback, among others [45,46].

#### 5.1.3. Create a Minimum Viable Product (MVP)

**Figure A2:**
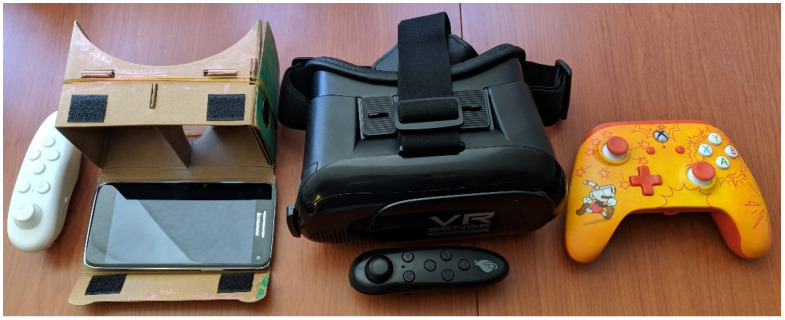
Technological prototypes used in the educational virtual reality environments.

Analyst, teachers, and programmer participants in this case study selected Google Cardboard [47,48]. As for the platform for the virtual reality environment, this is because it is compatible with any mobile device and can have connectivity with Bluetooth controls for the child’s interaction with the system. The MVP was a selected technological platform, intended to be accessible and low cost, so that they can be used by parents and teachers on their own mobile devices. As for the headset, Cardboard offers the option to build it from a diagram available on its website, which is made of cardboard, this can also be used so that the child can customize it to their liking or buy a plastic one at an affordable price as shown in Figure A2.

#### 5.1.4. Research and Learning

In this test stage, seven children with ADHD participated, covering the user profile defined by the multidisciplinary team. In a second test, the teachers recommended that regular children can also use virtual environments, so we included the participation of 9 children, giving a total of 16 children, all of them studying between their first and fourth year of elementary school.

**Figure A3:**
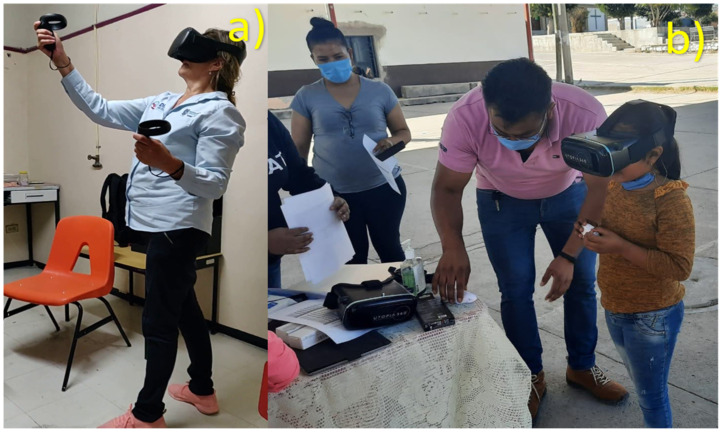
The first impressions on the use of virtual reality environments for teachers (**a**) and children (**b**).

In this iteration, the software tester proposed that teachers evaluate some virtual reality environments from some open educational repositories such as VIUR [49] and Education 3.0 [50]. One of the reasons for this was to explain to the teachers the purpose of using virtual reality environments as a support for school activities (see Figure A3). In addition, the software tester proposed that children use the virtual reality environments inside and outside the classroom (see Figure A3).

The members of the multidisciplinary team learned that there are a lot of online useful virtual reality environments such as VR Math app from Education 3.0 repository [50] and interactive theoretical learning from VIUR repository [49], but these applications do not cover the instructional design required for this case study. Then, a second iteration was necessary to propose new virtual environments with a mobile user interface that would integrate several functions to meet the learning needs of students with ADHD.

### 5.2. Second Iteration

A second iteration was conducted to propose a new educational virtual environment taking into account the user expectations of the previous iteration of the Lean UX model.

#### 5.2.1. Outcomes, Assumptions, and Hypotheses

The new virtual environment with a mobile user interface integrated several functions to cover basic learning needs for students with ADHD. This is an outcome coming from a consensual agreement in the previous iteration. In addition, the first assumption to consider here that it is possible to identify tasks as part of treatment according to the student profile with ADHD.

Once all the information from the evaluations of the children with ADHD and the recommendations of tasks as support to therapies were analyzed, the multidisciplinary team was given the tasks conforming the user profile that considers seven children with ADHD and nine regular children from different school grades as shown in Table 3. As the first assumption, the user profile presented in Table 3 is a product of the design made by teachers and psychologists in primary schools, which traditionally use this type of activities as a treatment in the classroom for children with ADHD and some cases in regular children to improve their attention and hyperactivity skills.

**Figure A4:**
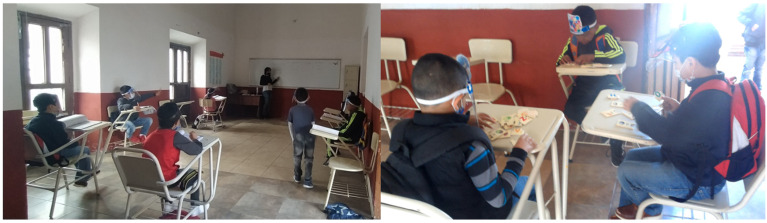
Teachers applying safety protocols such as the use of masks and disinfectant materials.

Figure A4 shows a blended education in the classroom with children with ADHD in the COVID-19 pandemic. As a second assumption, the teachers considered health protocols such as the use of masks and disinfectant materials. It is important to say that not only the children suffered changes in their processes, but also the work team that was responsible for conducting constant evaluations to follow up on the treatment carried out by the psychologists was limited to online or semi-presential sessions, which meant adopting new ways to continue working on achieving the objectives.

The identification of the user profile is one of the main activities that helped the multidisciplinary team to identify the tasks and appropriate platform on which the Attention-VR virtual reality environment is designed. For this purpose, an analysis was made of the results of various evaluations applied by teachers and psychologists to children with ADHD. Figure A4 shows some of the assessments used in the process of identification and treatment applied to children with ADHD.

We have considered as a hypothesis that a new virtual environment can support learning activities in enhancing learning experiences and playability. This is possible under a collaboration between different actors such as technologists, elementary school teachers, and students.

#### 5.2.2. Design It

Attention-VR has an instructional design (see Table 4) proposed by the multidisciplinary team so that the child can work the ADHD areas indicated in the identified user profile. These areas include location, attention, feedback, organization and sequence, motivation and stimulation, and rewards, among others. The instructional design of Attention-VR is presented in detail in Table 4.

Taking into account the previous instructional design given by pedagogue and teachers, the software designer then also proposed a user task design for the virtual environment Attention-VR in terms of the practical notation Concur Task Tree [51].

**Figure A5:**
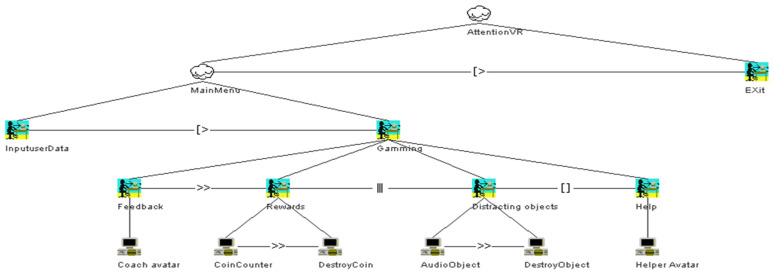
User task design related to the virtual environment Attention-VR.

According to the previous user task model (see Figure A5), once the user is registered, it is possible begin a new mission, then the user is required to follow some directions given by a coaching avatar. The Attention-VR environment is designed to help the user to focus on the instructions given by different avatars as well as to motivate them to accomplish the missions in a ludic manner. For this, the user can gain some coins by avoiding several distracting objects during the development of a mission. Note that a helper avatar is always enabled to advise the user.

#### 5.2.3. Create a Minimum Viable Product (MVP)

Considering the instructional design of previous sections, a new virtual educational environment called Attention-VR was developed in which the child must collect a series of coins that are part of a hidden treasure and find the pirate ship to leave the island. To achieve this objective, the child must solve challenges with the help of virtual assistants that indicate the instructions to follow in a visual and auditory way during the whole game to discover the way to the pirate ship. There are also distracting elements such as citizens that prowl around where there are coins and different objects as part of the virtual scenario. Each level has 3D virtual elements that always assist and provide feedback to the child in every situation presented within the virtual environment. The previous elements can be identified in the interactive prototypes for the virtual environment Attention-VR (see Table 5).

The user interface helps users to enter their identification data and it helps to have a good performance developing their activities. Activities include navigation in an immersive scenario with simulated 3D objects representing real-life objects with specific behavior. The user input contains the elements for the system to use various devices with which the child will interact during use.

#### 5.2.4. Research and Learning

**Figure A6:**
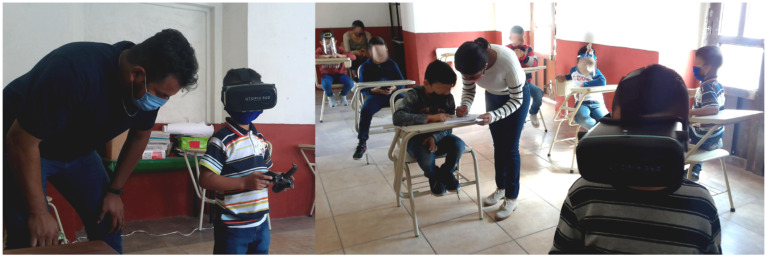
Use of virtual environment Attention-VR to support learning activities.

The research about effective use of the virtual educative environment Attention-VR involved the participation of teachers from all grades of an elementary school. The test required inserting the Attention-VR is into classes carried out under the online and semi presential modality with children with ADHD in order to provide assistance in the face of the limitations resulting from the COVID-19 pandemic. Teachers and children were brought together in the classroom under sanitary measures, which include mandatory use of masks and mouthpieces, a minimum distance of 1 m between people, and the use of antibacterial gel and alcohol to disinfect hands and objects, as shown in Figure A6.

**Figure A7:**
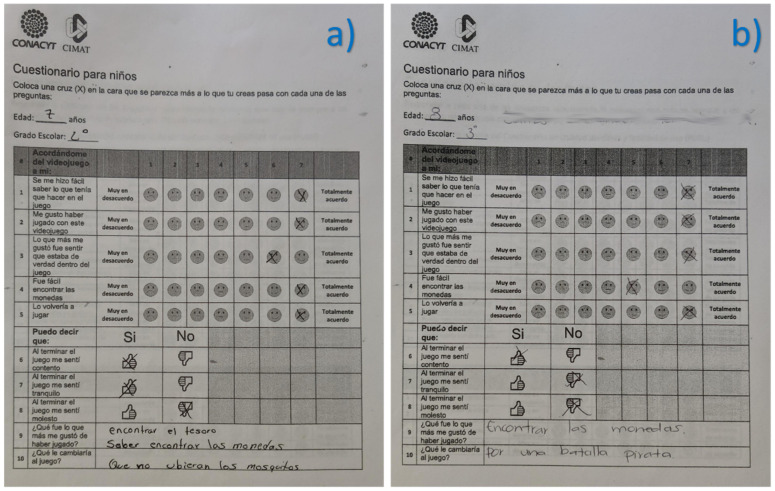
Assessment instruments Perception of use and satisfaction questionnaire applied to regular children (**a**) and ADHD children (**b**).

During the testing section, teachers applied an assessment instrument designed and approved by the multidisciplinary team to children with ADHD and regular children in order to learn about their perception of use and to evaluate the user experience. The design of this questionnaire was based on existing instruments in the literature such as the UEQ [52] and SUS [37] to measure the user experience and perception of the use of a product. With the help of psychologists and teachers, the questions were adjusted according to the context of the children, to ensure it was easy to understand and answer and could also be answered by a child with or without ADHD. Figure A7 presents some answers to the assessment instruments such as the perception of use and satisfaction questionnaire applied to children.

An analysis of the obtained results from these evaluations is presented and discussed in the following section.

## 6. Results

This section presents the results obtained from the production of a virtual reality environment Attention-VR in children with ADHD. As described in the previous section, the virtual reality environment was used by regular children from an elementary school. Therefore, it was an opportunity to collect information from the children, regarding the use, satisfaction, and performance when immersed in virtual reality.

### 6.1. Analysis of Reagents and Perception of Use and Satisfaction

The questionnaire designed to evaluate the perception of use and satisfaction after using Attention-VR is composed of 10 items (see Table A1), where the first five refer to the perception of the use of Attention-VR and use a Likert [53] scale from 1 to 7 (1 meaning very much in disagreement, 7 meaning totally in agreement).

Items 6 to 10 refer to satisfaction after using Attention-VR, 6 to 8 are answered with a Yes or No, and items 9 and 10 are open-ended questions to find out the child’s opinion and make improvements to the system.

Regarding the perception of use, the tendency in the children was to strongly agree that the system was always easy, and they knew what to do in the game (R1) and liked playing the game (R2). Regarding immersion, a high score was obtained (R3) and the children expressed their motivation to be immersed in a virtual reality environment. At the end of the game, they said it was not difficult to find all the coins (R4), and as to whether they would play the game again (R5) they strongly agreed to play again. Table 6 and Table 7 present the results of the surveys applied to children with ADHD and regular children who participated in the elementary education institution.

Regarding the items related to the feeling of being happy after having finished the game (R6), all the children said they felt happy. Only two children reported feeling excited after finishing the game (R7). As for the feeling of discomfort (R8), three children reported that at the beginning of the game they had to adapt to the immersion in the virtual environment, but later they reported discomfort when they wanted to play longer. Table 8 presents the results for these items.

### 6.2. Performance Analysis

**Figure A8:**
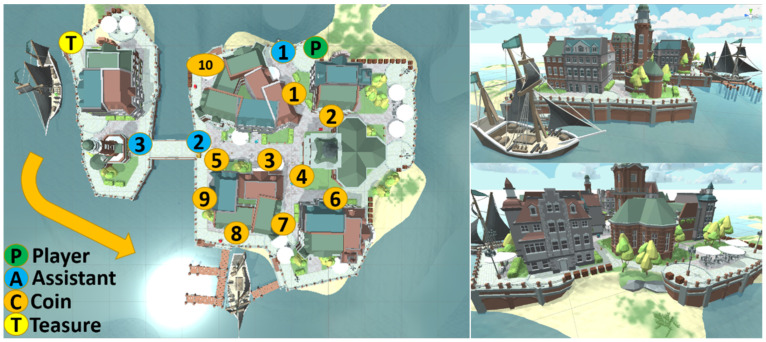
Map of the interactive environment Attention-VR.

The Attention-VR performance analysis is complemented with the map in Figure A8, which shows a distribution of the objects with which the child can interact. These objects include the Player (P) itself, the virtual assistants (A) represented by Avatars that assist the user at all times and can be consulted as many times as the child considers, the Coins (C) that are objects distributed all over the map and which the child has to make a strategy for their search and collection, and finally the Treasure (T) that allows the child to complete the activity and takes the child to the ship for a final virtual tour around the island. Attention-VR has a Firebase-based [54] storage mechanism to store the child’s information such as age and name. In addition, it records the time in minutes that the child collects each coin, as well as the order in which they collect them. It also records the number of times the child consulted a virtual assistant.

With this information, the multidisciplinary team was able to know the types of routes that are generated within the virtual environment and with this, the specialists and teachers were able to know the capacity that each child has to locate himself in time and space in a specific way. It was also possible to observe the way in which the child manages to pay attention to the stimuli and indications and the different decision making that the child carries out to sequence the task “catching coins” in small steps to structure his path, using the available resources fulfilling the objective in a friendly, concentrated, calm, and structured way to solve a problem.

Regarding the technical problems that occurred during the use of the system, of the 16 total children who used Attention-VR, 4 regular children had the application suddenly close when they were using it and also only a few items were recorded due to the lack of internet connection, so they were discarded for this analysis, leaving a sample of 12 children. In the case of the four children with technical problems, they made a second attempt to complete the activities within the system, but no performance information was obtained due to the lack of internet. It is worth mentioning that all 16 children answered the questionnaire of perception of use and satisfaction.

**Figure A9:**
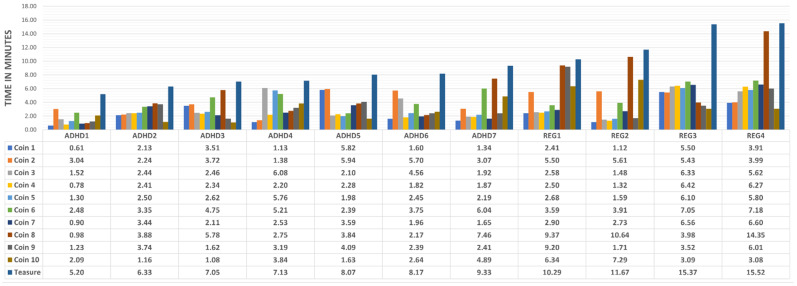
Time in minutes by children to solve Attention-VR, performance data obtained from the Firebase database.

The performance analysis obtained allowed us to know the total time in minutes that they remained within the virtual environment, the order of the coins obtained, and how many times they resorted to the help of one of the three assistants to obtain instructions within the game. Figure A9 presents a summary of the times obtained for each of the 12 children.

## 7. Discussion

This work obtained a satisfactory experience in using Lean UX to develop VR educational resources for the support of learning needs for ADHD students at elementary school. Due to the COVID-19 pandemic, teachers have been required and motivated to use technology inside and outside the classroom. This context helps to apply Lean UX better than the traditional approach, as teachers have proposed that covering different learning strategies allow to attract attention and increase the study concentration required for children with ADHD.

The use of Virtual Reality Environments aligned with Lean UX techniques was a key component to support learning activities for ADHD students. A second iteration was conducted to propose a new educational virtual environment taking into account the user expectations of the previous iteration of the Lean UX model. In general, the students were attracted to the idea of using the VRE as a new ludic manner to learn. The multidisciplinary and collaborative team was present in every iteration.

The collaborative and multidisciplinary work lets us focus on quick solutions according to the context of the elementary education user. The products have been designed and tested very quickly by the user such as teachers or students. Among the advantages of the proposed method, iterations offer the multidisciplinary team the ability to analyze and generate minimal products that can be tested by always considering the user and adjusting to their learning needs. So, this is continued in future iterations under a user-centered approach.

## 8. Conclusions and Future Work

This work advocates the production of virtual reality environments under the Lean UX model to design and create user experiences. These proposed virtual environments can be a support to the monitoring that basic education teachers do in school activities for children with ADHD. Due to the COVID-19 pandemic, they have had to adapt the classroom and continuous classroom work to semi-presential and online work. This work shows the benefits of the Lean UX model, which allows the production of virtual environments to be profitable so that each decision can be validated before starting work. The collaborative and multidisciplinary work ensures there is a focus on quick solutions according to the context of the elementary education user. Being iterative, the research and design progresses quickly allowing for the generation of minimal products that can be tested with users. Finally, the current user-centered approach allows knowing the users and their learning needs in each of its phases. A case study is presented in which all health measures were taken to produce and test the Attention-VR virtual reality environment with children with ADHD and regular children in different grades of basic education. The results obtained from the evaluation of the perception of use and satisfaction showed positive preliminary results and very good acceptance of Attention-VR. As future work, we are working on the production of new virtual reality environments under the Lean UX model that can be available on an open platform so that other institutions can use them and adapt them to their needs during the pandemic. In addition, other topics used by basic education teachers, such as mathematics and Spanish, among others, can be studied in depth. It also seeks to form new multidisciplinary teams to identify new needs of elementary education students and integrate new mechanisms for evaluating the user experience such as UQE, SUS, and AttrakDiff, among others. Finally, we are working on improving the platform to make it as accessible and available as possible to teachers, parents, and children, as well as incorporating mechanisms to capture as much information as possible for more detailed performance analysis.

## Figures and Tables

**Table 1:** Total children in elementary education order by scholar grade.

Scholar Grade	Regular Children	ADHD	Asperger	Intellectual Disability	Subtotal
1	21	2	1	0	24
2	20	3	0	0	23
3	14	2	1	5	22
4	19	1	0	2	22
5	9	0	0	3	12
6	9	0	0	2	11
Total	92	8	2	12	114

**Table 2:** Initial instructional design for the Attention-VR virtual reality environment.

Area	Instruction
Location	The child is placed in both time and space in a specific way.
Attention and feedback	The child identifies specific important objects within the immersive environment and discards those that represent a distraction. At each moment he/she obtains feedback to accomplish the task.
Organization and sequence	Small steps to accomplish within the virtual environment are indicated to accomplish a goal.
Motivation and structuring	Instructions are presented through elements such as audio, animations, and avatars so that the child can continue with the task.
The balance between demand and motivation	That the child is able to stay motivated in the game and at the same time is required to take a final step to achieve it. Without exceeding the demand since the child may lose motivation completely if he/she feels frustrated.
Reward and satisfaction	That the child feels satisfaction for having achieved the goal, leaving aside distractions, and reaching the happy ending, the final reward is represented in the form of visual and auditory stimulus.

**Table 3:** User profile of children with ADHD and regular children at elementary school level.

#	Scholar Grade	Age	Evaluation	Tasks as Treatment Support
ADHD			Attend to areas of ADHD related to location, attention, organization and sequence, motivation and structure, instruction	Instruction tracking Find differences in images Memory games Construction games Labyrinths Move-in slow motion Word search
2	1	6		
2	2	7		
2	2	8		
1	1	10		
Regular				
9	2	7		

**Table 4:** Extended instructional design for a new virtual environment called Attention-VR.

Action	Instruction	ADHD Area	Goal
Welcome to the game	Hello adventurer, welcome to the Magic City of 2020	Location	The child is placed in time and space in a specific way
Game instructions	Hello adventurer, first you must find the coins that appear on the road	Attention	Game instructions
Begin to catch coins. Distracting elements appear, but you are not yet instructed to concentrate	One coin, very good, go for the rest! two coins, three coins	Attention and positive feedback	The child pays attention to the coin, discarding the other dolls that appear with a distracting factor, without asking him to concentrate, he only feeds back when he catches the first three coins
Presenting elements: Some correspond to the coins and the others are only distracting visual stimuli as elements of the atmosphere	Focus on your goal and go for the coin. Four coins!	Organization and sequence	Sequence the task “catching coins” in small steps to achieve the structure of the task
Once the first coin is obtained (or simultaneously) you will hear an emotional sound and perhaps a motivational message	Five coins, very good, come on you can, you only have 6 more coins to find!	Motivation and structuring	That the child recognizes with the audio that he or she is doing what is expected. Short instruction to keep moving forward
Intermediate instruction on how to get to the end	Hello adventurer to enable the bridge you must find the 10 hidden coins, come on! you can do it	Intermediate Instruction	Let the child remember that to get to the end, he needs the 10 coins
Coins with auditory stimuli continue to appear every time one is captured. It feeds back and invites you to continue to concentrate	Eight coins, focus on your goal and look for the coin, nine coins	Attention and positive feedback	The child pays attention to the coin, listening to the number of coins it has, so he knows he is almost at the end
The child has achieved the 10 coins, he is motivated to continue and at the same time, he is asked to go through a bridge to get out of the city	Ten coins, very well-done adventurer, now I invite you to pass by the bridge	The balance between demand and motivation	That the child is able to continue motivated in the game and at the same time, it is required to take one last step to achieve it. Without exceeding the demand since the child can lose motivation completely if he feels frustrated
The child will find a treasure at the end of the bridge when arriving at the boat	Hello, adventurer find the treasure to get to the pirate ship, you can make it!	Motivation and Stimulation	Let the child know that he or she is already in the home stretch, his or her motivation continues, and the reward for his or her effort is in finding the treasure
End of the mission accompanied by fanfare	Congratulations on the successful completion of the mission!	Reward and satisfaction	The child feels satisfaction for having achieved the objective, leaving aside distractions, and arriving at the end are happy, the final reward is auditory stimulus when listening to the fanfares and rockets and cannons, in addition, visual stimulus when seeing the green confetti and stars

**Table 5:** Interactive prototypes for the virtual environment Attention-VR.

Item	Description	Interactive Prototypes
Mobile interface	The mobile interface allows capturing the child’s basic information to keep track of his activity within Attention-VR	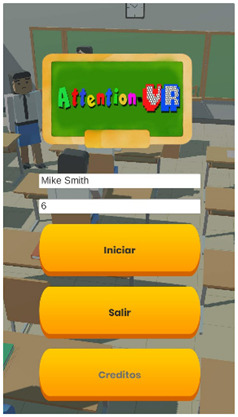
Helper avatars	Some avatars can be consulted and provide instructions regarding the activity to be performed, offer feedback information, remind the user of the steps to follow to accomplish the task, etc.	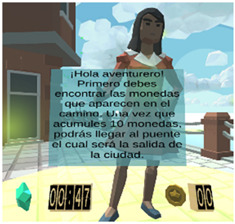
Distracting objects	These are 3D objects that can be animated or static, such as enemies, dynamic objects that move through the scenario but do not have any functionality related to the task to be performed.	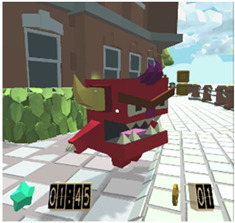
Rewards	These are animated or static 3D objects that can refer to the partial or total achievement of a task	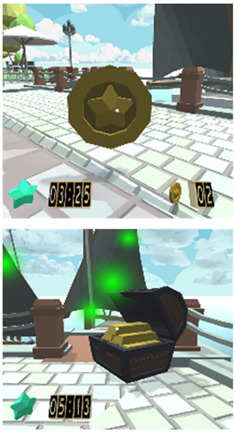
Feedback	These are multimedia elements within the virtual environment such as audio that transmits indications about the task, motivational phrases during the execution time, clues, and key tips for the correct resolution of the task, motivational phrases during the execution time, clues, and key tips for the correct resolution of the task	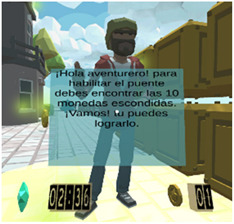

**Table 6:** Perception of use and satisfaction reagents from the assessment instrument applied to children who used Attention-VR.

General Data				Reagents According to Likert Scale				
Children	Name	Grade	Age	R1	R2	R3	R4	R5
ADHD	AD1	2	7	5	7	7	6	7
	AD2	1	6	7	7	7	5	7
	AD3	2	7	5	7	7	7	7
	AD4	1	6	7	7	7	7	4
	AD5	4	10	7	7	7	7	7
	AD6	3	8	7	7	7	5	7
	AD7	3	8	7	7	7	7	7
Regular	REG1	2	7	6	6	7	6	6
	REG2	2	7	7	7	7	4	7
	REG3	2	7	7	7	6	7	7
	REG4	2	7	7	7	6	7	7
	REG5	2	7	7	7	7	4	7
	REG6	2	7	7	6	7	7	7
	REG7	2	7	7	7	6	5	7
	REG8	2	7	7	7	7	6	7
	REG9	2	7	7	7	6	7	7

**Table 7:** Frequency analysis of the reagents of the evaluation of the perception of use and satisfaction.

General Data	Reagents According to Likert Scale					Total	%
Item Response	R1	R2	R3	R4	R5		
Strongly disagree	0	0	0	0	0	0	0%
Disagree	0	0	0	0	0	0	0%
Somewhat disagree	0	0	0	0	0	0	0%
Neither disagree nor agree	1	0	0	2	1	4	5%
Somewhat agree	2	0	0	3	0	5	6%
Agree	1	2	4	3	1	11	14%
Strongly agree	12	14	12	8	14	60	75%
Sum	16	16	16	16	16	80	100%

**Table 8:** Frequency analysis of R6 to R8 reagents.

General Data	Reagent			Total	%
Item Response	R6	R7	R8		
Yes	16	14	3	33	69%
No	0	2	13	15	31%
Sum	16	16	16	48	100%

## Data Availability

Not applicable.

## Supplementary Materials

Video online available at: https://youtu.be/xNhWVxR7-oU.

## Appendix A

**Table A1:** Reagents from the questionnaire used to evaluate the perception of use and satisfaction.

Section	Reagents
Perception	It was easy for me to know what to do in the game.
	I liked playing this video game.
	What I liked the most was feeling like I was really inside the game.
	It was easy to find the coins.
	I would play it again.
Satisfaction	At the end of the game, I felt happy.
	At the end of the game, I felt calm.
	At the end of the game, I felt annoyed.
	What did I like most about playing the game?
	What would you change about the game?
